# Evaluation of pleural fluid parameters related to cardiac diseases in cats

**DOI:** 10.14202/vetworld.2021.2238-2243

**Published:** 2021-08-27

**Authors:** Nutcha Chobsuk, Panita Pongprasit, Pralphun Puangploy, Monnakarn Bunnag, Luksika Keerativitayanant, Sirilak Disatian Surachetpong

**Affiliations:** Department of Veterinary Medicine, Faculty of Veterinary Science, Chulalongkorn University, Bangkok, Thailand

**Keywords:** cardiac disease, cat, pleural effusion, protein concentration

## Abstract

**Background and Aim:**

Cardiac disease is common in cats, and concurrent pleural effusion can worsen the prognosis. However, the definitive diagnosis of feline cardiac disease by echocardiography is limited in some areas. This study was designed to determine the indicators from fluid analysis obtained from pleural effusion to help diagnose cardiac disease in cats.

**Materials and Methods:**

A retrospective study was conducted. Data of 61 cats with pleural effusion were retrieved. The cats were divided into two groups: Cardiac disease (n=34) and non-cardiac disease (n=27) groups. Sex, neuter status, breed, age, weight, physical findings, fluid analysis results, radiographic findings, echocardiographic findings, and the presence of other diseases or conditions were recorded.

**Results:**

The Chi-square test showed the relationship between cardiac disease and the presence of abnormal heart sounds (p=0.0003), the N-terminal pro-B-type natriuretic peptide-positive result from serum samples (p=0.046), the presence of arterial thromboembolism (p<0.00001), and the presence of radiographic evidence of cardiomegaly and pulmonary edema (p=0.0002 and 0.001, respectively). The Mann–Whitney U-test showed a significant difference in protein concentration and specific gravity between the cardiac and non-cardiac disease groups (p=0.012 and 0.009, respectively). The decision tree classification method showed that protein concentrations of ≤3 g/dL were related to cardiac disease (sensitivity, 41.2% and specificity, 92.6%).

**Conclusion:**

The protein concentration in the pleural fluid may be used to distinguish cardiac and non-cardiac diseases in cats.

## Introduction

Cardiac disease commonly occurs in cats, especially cardiomyopathy, a group of myocardial diseases that can cause cardiac function and structure abnormalities. The etiology of cardiomyopathy in cats is mostly unknown, and the disease is potentially life-threatening. Cardiac disease is a common cause of death in cats [1]. The disease can be categorized into primary or idiopathic cardiomyopathy and secondary cardiomyopathy [2]. Primary cardiomyopathy consists of hypertrophic cardiomyopathy, restrictive cardiomyopathy, dilated cardiomyopathy, arrhythmogenic cardiomyopathy,and unclassified ­cardiomyopathy. Cardiomyopathy caused by other abnormalities, for example, hyperthyroidism, systemic hypertension, and acromegaly are considered secondary cardiomyopathies. The diagnostic test of choice for cardiomyopathy in cats is echocardiography [2]. As mentioned previously, feline cardiac disease may occur with a wide range of severity, from subclinical to unmanageable heart failure and death.Concurrent pleural effusion can worsen the prognosis [3]. The diagnosis is potentially challenging and is essential to provide early diagnosis and treatment. However, diagnosing cardiac disease using echocardiography, which is considered the diagnostic test of choice for detecting cardiac disease, is limited in some areas due to a lack of resources and experienced sonographers.

Pleural fluid is normally found in a small volume within the pleural space, lubricating movement of intrathoracic structures during respiration. Starling’s forces conduct pleural fluid formation and drainage [4]. Pleural effusion occurs when the factors determining Starling’s forces are altered to increase fluid formation or decrease fluid absorption [5]. The presence of pleural effusion may occur secondary to increased capillary hydrostatic pressure in the systemic and pulmonary circulation when the heart is no longer able to pump out the volume of blood effectively. Pleural effusion may present from other factors, including hypoalbuminemia, inflammation of the pleura, and lymphatic obstruction. Other concurrent diseases, for example, hyperthyroidism, feline leukemia virus (FeLV) infection, and lymphoma, may also influence pleural effusion development [6]. Studies have shown that the most common cause of pleural effusion in cats was congestive heart failure [3,7].Pleural effusion can be detected from clinical signs and physical findings and is simply confirmed by thoracocentesis or thoracic radiography [6]. Thoracocentesis should be performed as an initial treatment to stabilize the patient, followed by performing diagnostic methods using pleural fluid samples, for example, fluid analysis, which may help diagnose the underlying etiology. Effusions are classified into transudate, modified transudate, and exudate, according to cytological and biochemical criteria. Alteration of capillary hydrostatic pressure caused by congestive heart failure is associated with transudates and modified transudates, which are long-standing transudates [6]. The association between fluid types and underlying etiologies is less well defined, and the overlap between fluid analysis patterns is often found according to classifying factors,including total protein, total nucleated cell count (TNCC), and specific gravity. A study has shown that cats with cardiac disease had significantly lower protein concentrations and nucleated cell counts in the effusion than those with other diseases, such as feline infectious peritonitis (FIP), pyothorax, and neoplasia [7]. However, the cutoff values have not been developed.

As echocardiography is unavailable in every animal hospital, defining indicators from pleural fluid analysis may help distinguish feline cardiac diseases from other diseases. This study was designed to determine indicators from the fluid analysis that can be used to differentiate cardiac diseases from non-cardiac diseases in cats.

## Materials and Methods

### Ethical approval

The study design was a retrospective study. Ethical approval was not necessary.

### Study design, period and location

A retrospective study was conducted. Electronic medical records were retrieved from July 11, 2014, to August 27, 2020. Data were used from the Small Animal Hospital, Faculty of Veterinary Science, Chulalongkorn University, Thailand. Furthermore, signalment, clinical signs, laboratory findings, and disease diagnoses were recorded.

### Animals

Data on 352 client-owned cats that underwent thoracocentesis at the Department of General Medicine and the Cardiology Unit, Small Animal Hospital Faculty of Veterinary Science, Chulalongkorn University, were collected from July 11, 2014,to August 27, 2020. Of the 352 cats, 38 (10.79%) had incomplete recorded data. Moreover, 253 (71.87%) did not have pleural fluid analysis and/or echocardiographic results. Only 61 (17.32%) of the 352 cats had complete data for analysis.

All 61 cats underwent thoracocentesis, pleural fluid analysis, and echocardiography. Echocardiography was the gold standard method for detecting cardiac disease in this study. Moreover, 34 and 27 cats were diagnosed with cardiac disease and non-cardiac disease, respectively. The types of diseases were noted. Data on the fluid analysis reported by the laboratory of the Department of Pathology, Faculty of Veterinary Science, Chulalongkorn University, were recorded. Fluids were categorized into transudate (specific gravity of <1.012, protein level of <2.5 g/dL, and TNCC of <1500 cells/μL), modified transudate (specific gravity of <1.012-1.020, protein level of <2.5-5.0 g/dL, and TNCC of <1500-7000 cells/μL), and exudate (specific gravity of more than 1.020, protein less than of more than 3 g/dL, and TNCC of more than 7000 cells/μL) [5].

### Statistical analysis

Study variables included sex, neuter status, breed, age, weight, heart sound, lung sound, the result of the point-of-care test kit of N-terminal pro-B-type natriuretic peptide (NT-ProBNP) from serum samples, fluid analysis (i.e., TNCC, cytology, protein concentration, specific gravity, and bacterial culture), total thyroxine (T4) level, chest radiographic findings, echocardiographic findings, and the presence of concurrent diseases or conditions, including systemic hypertension (i.e., systolic arterial blood pressure of more than 140 mmHg) [8], hyperthyroidism (total T4>4 μg/dL), arterial thromboembolism (ATE), chronic kidney disease, and common feline viral infection (e.g., FeLV, feline immunodeficiency virus [FIV], and FIP). FeLV and FIV were diagnosed using FeLV antigen and FIV antibody enzyme-linked immunosorbent assay test kits. FIP was suspected using signalment, history,clinicopathological abnormalities, and laboratory results, such as the albumin-to-globulin ratio and Rivalta test.

Microsoft Excel 2019 (version 16.0, Microsoft Corporation, Washington, USA) was used to perform descriptive statistical analysis. Categorical data were presented as a percentage, and variables were compared using the Chi-square test. p≤0.05 was considered statistically significant.

Statistical Package for the Social Sciences (IBM SPSS software version 27.0.1, SPSS Inc., Chicago, Ill., USA) was used to perform all statistical analyses. Quantitative data were presented as medians with 25^th^-75^th^ interquartile ranges.The Mann–Whitney U-test was used to compare variables between groups. p≤0.05 was considered statistically significant.

A decision tree model was created using R program (R version C5.0, R core team, Vienna, Austria) to employ a recursive binary partitioning algorithm, splitting the sample into partitioning variables with the strongest association with the response variable [9]. The top node is the finest node for classification, whereas the other features in the nodes appear in descending order of importance. Only features contributing to the classification appear in the decision tree.

## Results

Data of 61 cats were included in the study.The cats were divided into the cardiac (n=34) and non-cardiac (n=27) disease groups based on echocardiographic results. The population characteristics of 61 cats and the results of the Mann–Whitney U-test on quantitative data are summarized in Table-1.

**Table-1:** Population characteristics of 61 cats with and without cardiac disease.

Variables	Category	Cardiac (n=34)	Non-cardiac (n=27)	p-value
Sex	Male	14 (41.17)	11 (40.74)	0.973
	Female	20 (58.83)	16 (59.26)	
Status	Sterile	22 (64.7)	15 (37.03)	0.564
	Intact	9 (29.4)	11 (40.74)	
	Unknown	3 (8.82)	1 (3.7)	
Breed	DSH	27 (79.41)	24 (88.89)	0.682
	Persian	3 (8.82)	1 (3.7)	
	Others	4 (11.77)	2 (7.41)	
Age (years)		8 (2-12)	6 (2.25-11)	0.629
Weight (kg.)		3.7 (3-4.7)	3.79 (3.3-4.66)	0.469
Heart sounds	Normal	12 (35.3)	22 (81.5)	0.0003
	Abnormal (murmur/gallop/muffle)	22 (64.7)	5 (18.5)	
Lung sounds	Normal	8 (23.53)	7 (25.9%)	0.829
	Abnormal (dull/increase/decrease/crackle)	26 (76.47)	20 (74.1)	
NT-proBNP	Positive	5 (55% of 9 cats)	1 (11.1% of 9 cats)	0.046
	Negative	4 (45% of 9 cats)	8 (88.8% of 9 cats)	
	Non-test	24 (70.6)	18 (66.7)	
SH	Positive	7 (43.7% of 16 cats)	3 (30% of 10 cats)	0.483
	Negative	9 (56.2% of 16 cats)	7 (70% of 10 cats)	
	Non-test	18 (52.9)	17 (62.9)	
ATE	Positive	2 (8.3% of 24 cats)	0	<0.00001
	Negative	22 (91.7% of 24 cats)	15 (100% of 15 cats)	
	Non-test	10 (29.4)	12 (44.4)	
Concurrent disease	Viral infectious disease	11 (32.35)	14 (51.8)	0.189
	Hormonal disease	4 (11.7)	2 (7.4)	
	Tumor	4 (11.7)	10 (37)	
	Chronic kidney disease	11 (32.35)	7 (25.29)	
	Other disease	0	2 (7.4)	
Pleural fluid analysis				
TNCC (cells/µL)		725 (256.25-1237.5)	1100 (187.5-8031.25)	0.135
Protein (g/dL)		3.6 (3-4.7)	4.4 (3.9-5.4)	0.012
Specific gravity		1.022 (1.01825-1.02775)	1.027 (1.023-1.0305)	0.009
Effusion type	Transudate	9 (26.4)	3 (11.1)	0.391
	Modified transudate	22 (64.7)	16 (59.2)	
	Exudate	3 (8.8)	8 (29.6)	
Bacterial culture	No growth	22 (64.7)	22 (81.4)	
	Growth	4 (11.7)	4 (14.8)	
	Non-culture	8 (23.5)	1 (3.7)	
Radiography	Cardiomegaly	18 (52.9)	2 (7.4)	0.0002
	Pulmonary edema	15 (44.1)	2 (7.4)	0.001
	Ascites	10 (29.4)	4 (14.8)	0.178

Categorical data presented as number of cats, percent (%) and compared between two groups using Chi-square test. Quantitative data were all non-normal distribution. It expressed as medians (25^th^-75^th^ interquartile range) and compared between two groups using the Mann–Whitney U-test. NT-proBNP=Serum N-terminal pro-B-type natriuretic peptide; SH=Systemic hypertension; ATE=Arterial thromboembolism; TNCC=Total nucleated count cells; kg.=Kilogram

The Chi-square test on categorical data showed that the presence of abnormal heart sounds (p=0.0003), NT-proBNP-positive results from serum samples (p=0.046), the presence of ATE (p<0.00001),and the presence of radiographic evidence of cardiomegaly and pulmonary edema (p=0.0002 and 0.001, respectively) were related to cardiac disease in cats.

**Figure-1:**
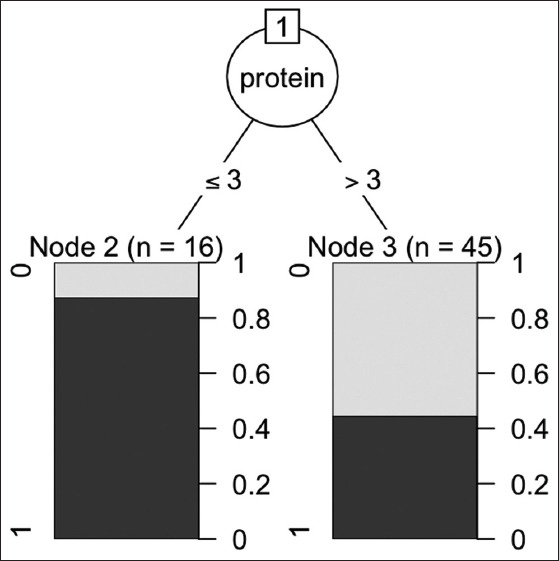
The result of decision tree classification methods C5.0, the first group represents a percentile of cats that have protein concentration ≤3 g/dL and >3 g/dL in the next group. The data are only two classes (cardiac disease=1 and non-cardiac disease=0).

The Mann–Whitney U-test and descriptive analysis of quantitative data showed a significant difference in protein concentration and specific gravity between the cardiac and non-cardiac disease groups (p=0.012 and 0.009, respectively). The decision tree classification method showed that the decision tree has one leaf (classification) node. The root node (placed at the top of the tree) is protein concentration. The root node was split into two groups at a protein concentration of 3 g/dL (Figure-1).

The decision tree classification method showed that protein concentrations of ≤3 g/dL were related to cardiac disease (sensitivity, 41.2%;specificity, 92.6%; positive predictive value [PPV], 87.5%;and negative predictive value [NPV], 55.6%).

The decision tree showed that specific gravity could not be used to differentiate cats with cardiac disease from those with non-cardiac disease. Specific gravity of ≤1.027 g/dL had 62.9% sensitivity,70.5% specificity, 62.9% PPV,and 70.5% NPV for differentiating the cardiac and non-cardiac disease groups.

## Discussion

This study found that the protein concentration and specific gravity of pleural fluid were associated with cardiac diseases in cats. These parameters may help distinguish cardiac diseases from non-cardiac ones in cats.A study has demonstrated that cardiac disease was the most common cause of pleural effusion in cats followed by FIP and neoplasia [7]. Another study has found that cardiac disease was the most common cause of pleural effusion followed by neoplasia [10]. This finding contrasts with an older study demonstrating that cardiac disease was the less common cause of pleural effusion, whereas neoplasia and pyothorax were the top two common causes of pleural effusion in cats [11]. These findings suggest that the causes of pleural effusion vary depending on geographical differences and diagnostic possibilities.

This study showed an overlap of age between cats with and without cardiac disease, suggesting that age is not a suitable parameter for distinguishing cats with cardiac disease from those without cardiac disease. This finding agrees with the previous studies that have demonstrated age overlaps in cats with pleural effusion from several causes [7,10]. However, age may help determine causes of pleural effusion secondary to some diseases, such as FIP and lymphoma, which are found mainly in young cats [7,10].

Cats with cardiac disease may have a low body temperature secondary to poor perfusion [12]. A study has shown that a low body temperature is a good parameter to distinguish cats with cardiac disease from those without cardiac disease [7,10]. However, heart rate and respiratory rate could not be used to differentiate cats with cardiac disease from those without cardiac disease [10]. This study showed that abnormal heart sounds (i.e., murmurs/gallops/muffles) helped discriminate cats with cardiac disease from those without cardiac disease. The American College of Veterinary Internal Medicine consensus statement guidelines for the classification diagnosis and management of cardiomyopathies in cats suggest that cats with murmur/gallop heart sounds or arrhythmia are more likely to have cardiac disease and need further investigations for diagnosis [2].

The alteration of hydrostatic pressure secondary to congestive heart failure can lead to pleural effusion [13]. Usually, the fluid leaking from an increased hydrostatic pressure is low-protein fluid with low specific gravity. In contrast, exudates, which are fluids with high protein and high specific gravity, usually come from the alteration of vascular permeability [14]. This study found that the protein concentration (p=0.012) and specific gravity (p=0.009) of pleural fluid were significantly related to cardiac disease in cats. The median protein concentration of cats in the cardiac disease group was lower than that in the non-cardiac disease group. Fluid with low protein and specific gravity suggests transudates commonly caused by congestive heart failure [6]. In human medicine, a protein cutoff level of 3.0 g/dL has an accuracy of approximately 90% to differentiate transudates from exudates [15]. This cutoff is also used in veterinary medicine [5]. The results of the decision tree classification method suggest that cats with pleural fluid protein concentration of ≤3 g/dL were related to cardiac diseases (sensitivity, 41.2% and specificity, 92.6%). Due to the high specificity of the test, cardiac diseases should be suspected in cats with a pleural fluid protein concentration of ≤3 g/dL.

This study also found a difference in specific gravity between cats with and without cardiac disease. However, the difference is minimal. In addition, using specific gravity with the cutoff of 1.027 provided low sensitivity and specificity to differentiate cats with cardiac disease from those without cardiac disease. Therefore, the specific gravity of pleural fluid may not be a good indicator for distinguishing cats with cardiac diseases from and those with non-cardiac diseases in clinical practice.

Pleural fluid type is another indicator that may help diagnose cardiac diseases. Pleural fluid from cats with cardiac disease is usually transudate and modified transudate [6]. In this study, 67.4% and 26.4% of cats with cardiac disease had modified transudate and transudate, respectively. Only 8.8% of cats with cardiac diseases had exudative pleural fluid. Moreover, 5.8% of cats with cardiac disease with exudates had concurrent diseases (i.e., mediastinal lymphoma), and 2.9% of them did not have concurrent diseases. The cytologic findings of exudative fluid from cats with cardiac disease with and without concurrent diseases were similar and indistinguishable. To the authors’ knowledge, the incidence of exudative pleural ­effusion in cats with cardiac diseases has not been published. In human medicine, most patients with congestive heart failure and exudative effusions have an underlying cause for their exudate. Exudative pleural effusions secondary to solely congestive heart failure are rare [16]. According to a study in human medicine, underlying diseases should also be identified in cats with cardiac disease and exudative pleural fluid.

Besides congestive heart failure, several diseases and conditions can cause pleural effusion. Hyperthyroidism may induce cardiac abnormalities and cause transudate or modified transudate effusion [4]. FIP can cause massive vasculitis and result in fluid leakage and exudate accumulation [17]. Extreme hypoalbuminemia may lower systemic colloidal osmotic pressure sufficient to cause transudate accumulation [18]. Intrathoracic neoplasia may cause exudate effusion with neoplastic cells [4]. The characteristics of fluid are different depending on the underlying pathophysiological process of diseases. However, the overlap of fluid types between etiologies can sometimes be found. Therefore, fluid types alone cannot determine the exact cause of pleural effusion in every case. However, it may provide clues for diagnosis. Other clinical information and techniques, such as physical findings, radiographic findings, and NT-proBNP concentration, may be necessary to complete the diagnosis.

The Chi-square test showed that the presence of abnormal heart sounds, the presence of ATE, and NT-proBNP-positive results from serum samples were related to cardiac disease in cats, suggesting that these indicators may be used to support the diagnosis of cardiac disease in cats. A study has shown that the prevalence of cardiogenic ATE was higher than that of non-cardiogenic ATE [19]. Furthermore, ATE is one of the most common complications of cardiomyopathies in cats [2]. Therefore, we recommend further investigate cardiac diseases in every cat affected with ATE. Measuring NT-proBNP concentration in pleural fluid can differentiate cardiogenic causes from non-cardiogenic causes of effusion in cats [20,21]. However, the quantitative test of NT-proBNP is unavailable in Thailand now. A study has shown that a point-of-care test measuring NT-proBNP in diluted pleural effusion (1:1 pleural effusion to 0.9% saline solution) was suitable to distinguish cardiogenic causes from non-cardiogenic causes of pleural effusion. However, this method was not performed in pleural fluid samples in this study [22].

The study has some limitations due to its retrospective design, such as missing data. Another limitation was the small number of cats in each group that may affect the statistical analysis. In addition, the protein concentration in this study was measured using the supernatant after centrifugation of pleural fluid samples to avoid falsely high-protein concentrations [23]. Therefore, the results from this study cannot be used using uncentrifuged samples.

## Conclusion

This study demonstrates that the protein concentration from pleural fluid analysis is associated with cardiac diseases in cats. Therefore, pleural fluid protein concentration may be used as an indicator to evaluate the risk of cardiac disease in cats with pleural effusion before confirming the diagnosis using echocardiography, which is the gold standard method for diagnosing cardiac diseases in cats. However, protein concentration cannot be used as a standalone indicator for diagnosing cardiac diseases in cats. Other techniques, such as NT-proBNP measurements, radiography, and echocardiography, should be performed to confirm the presence of cardiac diseases.

## Authors’ Contributions

NC, PP, PPu, MB, and LK; Data collection, data analysis, and writing of first draft: SDS: supervision, data validation, and writing, review and editing. All authors read and approved the final manuscript.
